# Efficient Post-Shrinkage Estimation Strategies in High-Dimensional Cox’s Proportional Hazards Models

**DOI:** 10.3390/e27030254

**Published:** 2025-02-28

**Authors:** Syed Ejaz Ahmed, Reza Arabi Belaghi, Abdulkhadir Ahmed Hussein

**Affiliations:** 1 Department of Mathematics and Statistics, Brock University, St. Catharines, ON L2S 3A1, Canada; sahmed5@brocku.ca; 2 Department of Energy and Technology, Swedish University of Agricultural Sciences, P.O. Box 7032, 750 07 Uppsala, Sweden; reza.belaghi@slu.se; 3 Department Mathematics & Statistics, University of Windsor, Windsor, ON N9B 3P4, Canada

**Keywords:** variable selection, high-dimensional data, Cox proportional hazards model, LASSO, shrinkage estimation, sparse model, 62J12, 62J07, 62E20, 62F25

## Abstract

Regularization methods such as LASSO, adaptive LASSO, Elastic-Net, and SCAD are widely employed for variable selection in statistical modeling. However, these methods primarily focus on variables with strong effects while often overlooking weaker signals, potentially leading to biased parameter estimates. To address this limitation, Gao, Ahmed, and Feng (2017) introduced a corrected shrinkage estimator that incorporates both weak and strong signals, though their results were confined to linear models. The applicability of such approaches to survival data remains unclear, despite the prevalence of survival regression involving both strong and weak effects in biomedical research. To bridge this gap, we propose a novel class of post-selection shrinkage estimators tailored to the Cox model framework. We establish the asymptotic properties of the proposed estimators and demonstrate their potential to enhance estimation and prediction accuracy through simulations that explicitly incorporate weak signals. Finally, we validate the practical utility of our approach by applying it to two real-world datasets, showcasing its advantages over existing methods.

## 1. Introduction

High-dimensional data analysis, where the number of covariates frequently exceeds the sample size, has become a central research focus in contemporary statistics (see [1]). The applications of these methods span a broad range of fields, including genomics, medical imaging, signal processing, social science, and financial economics. In particular, high-dimensional regularized Cox regression models have gained traction in survival analysis (e.g., [2,3,4]), where these techniques help construct parsimonious (sparse) models and can outperform classical selection criteria such as Akaike’s information criterion [5] or the Bayesian information criterion [6].

The least absolute shrinkage and selection operation (LASSO) proposed by [7] remains one of the most popular approaches to high-dimensional regression, due to its computational efficiency and its ability to perform variable selection and parameter shrinkage simultaneously. Numerous extensions of LASSO, such as adaptive LASSO [8], elastic net [9], and scaled LASSO [10], have been developed to further refine estimation and prediction performance. In the context of Cox proportional hazards models, analogous methods—including the LASSO [4,11], the adaptive LASSO [12,13], and smoothly clipped absolute deviation (SCAD; [14])—have been widely examined. Interested readers may also consult [15,16,17,18] for recent advancements in high-dimensional Cox regression.

When p>n, the focus is often on accurately recovering both the support (i.e., which covariates have nonzero effects) and the magnitudes of the nonzero regression coefficients. Although many penalized inference procedures excel at identifying “strong” signals (i.e., coefficients that are moderately large and thus easily detected), they may fail in adequately accounting for “weak” signals, whose effects may be small but nonzero. To formalize this, one can divide the index set $\left\{1,\ldots ,p_{n}\right\}$ into three disjoint subsets as follows: $S_{1}$ for strong signals, $S_{2}$ for weak signals, and $S_{\mathrm{null}}$ for coefficients that are exactly zero. Standard estimation procedures that neglect weak signals risk introducing non-negligible bias, particularly when these weak signals are numerous.

In this paper, we tackle the bias induced by weak signals in high-dimensional Cox regression by adapting the post-selection shrinkage strategy proposed by [19]. Our key contribution is the development of a weighted ridge (WR) estimator, which effectively differentiates small, nonzero coefficients from those that are truly zero. We show that the resulting post-selection estimators dominate submodel estimators derived from standard regularization methods such as LASSO and elastic net. Moreover, under the condition $p_{n}=O\left(n^{\alpha }\right)$ for some α>0, we establish the asymptotic normality of our post-selection WR estimator, thereby demonstrating its asymptotic efficiency. Through extensive simulations and real data applications, we illustrate that our method achieves substantial improvements in both estimation accuracy and prediction performance.

The remainder of this paper is organized as follows. Section 2 presents the model setup and the proposed post-selection shrinkage estimation procedure. In Section 3, we outline the asymptotic properties of our estimators. Section 4 provides a Monte Carlo simulation study, while Section 5 reports the results of applying our methodology to two real data sets. We conclude in Section 6 with a brief discussion of possible future research directions.

## 2. Methodology

### 2.1. Notation and Assumptions

In this section, we state some standard notations and assumptions, used throughout the paper. We use bold upper case letters for matrices and lower case letters for vectors. Moreover, T denotes the matrix transpose and $I_{N}$ denotes the N×N identity matrix. Design vectors, or columns of X, are denoted by $X_{j},j=1,\cdots ,p_{n}$. The index set $M=\{1,2,\cdots ,p_{n}\}$ denotes the full model which contains all the potential variables. For a subset A⊂M, we use $\beta_{A}$ for a subvector of $\beta_{M}$ indexed by A, and $X_{A}$ for a submatrix of X whose columns are indexed by A. For a vector $v={\left(v_{1},\cdots ,v_{p_{n}}\right)}^{T}$, we denote ${\left||v|\right|}_{2}=\sqrt{\sum_{j=1}^{p_{n}}v_{j}^{2}}$ and ${\left||v|\right|}_{1}=\sum_{j=1}^{p_{n}}\left|v_{j}\right|$. For any square matrix A, we let $\Lambda_{\min}\left(A\right)$ and $\Lambda_{\max}\left(A\right)$ be the smallest and largest eigenvalues of A, respectively. Given a,b∈R, we let a∨b and a∧b denote the maximum and minimum of *a* and *b*. For two positive sequences $a_{n}$ and $b_{n}$, $a_{n}≍b_{n}$, if $a_{n}$ is in the same order as $b_{n}$. We use I(.) to denote the indicator function; $H_{\vartheta }\left(.;\Delta \right)$ denotes the cumulative distribution function (cdf) of a non-central $\chi^{2}$-distribution with ϑ degrees of freedom and non-centrality parameter Δ. We also use $\overset{D}{⟶}$ to indicate convergence in distribution.

Let $S\subset \{1,\cdots ,p_{n}\}$ be the set of the indices of nonzero coefficients, with s=|S| denoting the cardinality of S. We assume that the true coefficient vector $\beta^{*}={\left(\beta_{1}^{*T},\cdots ,\beta_{p_{n}}^{*T}\right)}^{T}$ is sparse, that is s<n. Without loss of generality, we partition the ($n\times p_{n}$)-matrix X as $X={\left(X_{S_{1}},X_{S_{2}},X_{S_{\mathrm{null}}}\right)}^{T}$, where $S_{1}\cap S_{2}\cap S_{\mathrm{null}}=⌀$, $S_{1}\cup S_{2}\cup S_{\mathrm{null}}=M$ and $S_{\mathrm{null}}=\left\{j:\beta_{0j}=0\right\}$. For two matrices $X_{S_{1}}$ and $X_{S_{2}}$, we define the corresponding sample covariance matrices by

(1) $$\begin{array}{cc} \Sigma_{S_{1}|S_{2}} & =\Sigma_{S_{1}S_{1}}-\Sigma_{S_{1}S_{2}}\Sigma_{S_{2}S_{2}}^{-1}\Sigma_{S_{2}S_{1}},\\ \Sigma_{S_{2}|S_{1}} & =\Sigma_{S_{2}S_{2}}-\Sigma_{S_{2}S_{1}}\Sigma_{S_{1}S_{1}}^{-1}\Sigma_{S_{1}S_{2}}. \end{array}$$

Let $V={\left(X_{S_{2}},X_{S_{\mathrm{null}}}\right)}^{T}$ be a $p_{n}-s_{1}$ submatrix of X. Then, another partition can be written as $X={\left(X_{S_{1}},V\right)}^{T}$. Let $M_{1}=I_{n}-X_{S_{1}}{\hat{\Sigma }}_{S_{1}S_{1}}^{-1}X_{S_{1}}^{T}$. Then, $V^{T}M_{1}V$ is a $\left(p_{n}-s_{1}\right)\times \left(p_{n}-s_{1}\right)$ dimensional singular matrix with rank $k_{1}\geq 0$. We denote $\varrho_{1}\leq \cdots \leq \varrho_{k_{1}}$ as all the $k_{1}$ positive eigenvalues of $V^{T}M_{1}V$.

### 2.2. Signal Strength Regularity Conditions

We consider three signal strength assumptions to define three sets of covariates according to their signal strength levels as follows [19]:

**(A1)**  There exists a positive constant $c_{1}$, such that $|\beta_{j}|≳c_{1}\sqrt{(\log p)/n}$ for $\forall j\in S_{1}$;
**(A2)**  The coefficient vector β satisfies $\left|\right|\beta_{S_{2}}{\left|\right|}_{2}^{2}∼O\left(n^{\tau }\right)$ for some 0<τ<1, where $\beta_{j}\neq 0$ for $\forall j\in S_{2}$;
**(A3)**  $\beta_{j}=0$, for $\forall j\in S_{\mathrm{null}}$.

### 2.3. Cox Proportional Hazards Model

The proportional hazards (PH) model introduced by [20] is one of the most commonly used approaches for analyzing survival data. In this model, the hazard function for an individual depends on covariates through a multiplicative effect, implying that the ratio of hazards for different individuals remains constant over time. We consider a survival model with a true hazard function $\lambda_{0}\left(t\;|\;X\right)$ for a failure time *T*, given a covariate vector $X={\left(X_{1},\ldots ,X_{p}\right)}^{T}$. We let *C* denote the censoring time and define Y=min(T,C) and $\delta =I\left(T\leq C\right)$. Suppose we have *n* i.i.d. observations ${\left\{\left(Y_{i},\delta_{i},X_{i}\right)\right\}}_{i=1}^{n}$ from this true underlying model, where $X={\left(X_{1},\ldots ,X_{p}\right)}^{T}$ represents the n×p design matrix.

The PH model posits that the hazard function for an individual with covariates X is

(2) $$\lambda \left(t\;|\;X\right)\;=\;\lambda_{0}\left(t\right)\;\exp\;\left(X^{T}\beta \right),$$

where $\beta ={\left(\beta_{1},\ldots ,\beta_{p}\right)}^{T}$ is the vector of regression coefficients, and $\lambda_{0}\left(t\right)$ is an unknown baseline hazard function. Because $\lambda_{0}\left(t\right)$ does not depend on X, one can estimate β by maximizing the partial log-likelihood

(3) $$l\left(\beta \right)\;=\;\sum_{i=1}^{n}\delta_{i}\;\left(x_{i}^{T}\beta \right)\;-\;\sum_{i=1}^{n}\delta_{i}\;\log\;\left(\sum_{j\in R(t_{i})}\exp\;\left(x_{j}^{T}\beta \right)\right),$$

where $\delta_{i}=I\left(T_{i}\leq C_{i}\right)$ and $R\left(t_{i}\right)=\left\{\;j:T_{j}\geq t_{i}\right\}$ is the risk set just prior to $t_{i}$. Maximizing l(β) in (3) with respect to β yields the estimator $\hat{\beta }$ for the regression parameters.

### 2.4. Variable Selection and Estimation

Variable selection can be carried out by minimizing the penalized negative log-partial likelihood as follows:

(4) $$-\;l\left(\beta \right)\;+\;\sum_{j=1}^{p_{n}}P_{\lambda }\left(\beta_{j}\right),$$

where $P_{\lambda }\left(\beta_{j}\right)$ is a penalty function applied to each component of β, and λ is a tuning parameter that controls the magnitude of penalization. We consider the following two popular methods:

1. **LASSO.** The LASSO estimator follows (4) with an $L_{1}$-norm penalty,
  $${\mathrm{Pen}}_{\lambda }\left(\beta_{j}\right)\;=\;\lambda \left|\beta_{j}\right|.$$
  As λ increases, this penalty continuously shrinks the coefficients toward zero, and some coefficients become exactly zero if λ is sufficiently large. The theoretical properties of the LASSO are well studied; see [21] for an extensive review.
2. **Elastic Net (ENet).** The Elastic Net estimator implements (4) with the combined penalty
  (5) $$P_{\lambda }\left(\beta_{j}\right)\;=\;\lambda \left(\alpha \;|\beta_{j}|\;+\;\left(1-\alpha \right)\;\beta_{j}^{2}\right),$$
  where 0≤α≤1. When α=1, this reduces to the LASSO, and when α=0, it becomes Ridge. Combining $L_{1}$ and $L_{2}$ penalties leverages the benefits of Ridge while still producing sparse solutions. Unlike LASSO, which can select *n* variables at most, ENet has no such limitation when $p_{n}>n$.

#### 2.4.1. Variable Selection Procedure for $S_{1}$ and $S_{2}$

We summarize the variable selection procedure for detecting the strong signals $S_{1}$ and the weak signals $S_{2}$.

**Step 1 (detection of $S_{1}$).** Obtain a candidate subset ${\hat{S}}_{1}$ of strong signals using a penalized likelihood estimator (PLE). Specifically, consider
  (6) $${\hat{\beta }}^{\mathrm{PLE}}\;=\;\arg\min_{\beta }\left\{-l_{n}\left(\beta \right)\;+\;\sum_{j=1}^{p_{n}}P_{\lambda }\left(\beta_{j}\right)\right\},$$
  where $P_{\lambda }\left(\beta_{j}\right)$ penalizes each $\beta_{j}$, shrinking weak effects toward zero and selecting the strong signals. The tuning parameter λ>0 governs the size of the subset ${\hat{S}}_{1}$.
**Step 2 (detection of $S_{2}$).** To identify ${\hat{S}}_{2}$, first solve a penalized regression problem with a ridge penalty only on the variables in ${\hat{S}}_{1}^{c}$. Formally,
  (7) $${\hat{\beta }}^{r}\;=\;\arg\min_{\beta }\left\{-l\left(\beta \right)\;+\;r_{n}\;{∥\beta_{{\hat{S}}_{1}^{c}}∥}_{2}^{2}\right\},$$
  where $r_{n}>0$ is a tuning parameter controlling the overall strength of regularization for variables in ${\hat{S}}_{1}^{c}$. We then define a post-selection weighted ridge (WR) estimator ${\hat{\beta }}^{\mathrm{WR}}$ by
  (8) $$\begin{array}{c} {\hat{\beta }}_{j}^{\mathrm{WR}}\;=\;\left\{\begin{array}{cc} {\hat{\beta }}_{j}^{r},\; & j\;\in \;{\hat{S}}_{1},\\ {\hat{\beta }}_{j}^{r}\;I\left(\left|{\hat{\beta }}_{j}^{r}\right|\;>\;a_{n}\right),\; & j\;\in \;{\hat{S}}_{1}^{c}, \end{array}\right. \end{array}$$
  where $a_{n}$ is a thresholding parameter. The set ${\hat{S}}_{2}$ is then
  (9) $${\hat{S}}_{2}\;=\;\left\{j\;\in \;{\hat{S}}_{1}^{c}\;:\;{\hat{\beta }}_{j}^{\mathrm{WR}}\neq 0,\;1\leq j\leq p\right\}.$$
  We apply this post-selection procedure only if $\left|{\hat{S}}_{2}\right|\;>\;2$. In particular, we set
  (10) $$a_{n}\;=\;c\;n^{-\kappa },\;0<\kappa \;\leq \;\frac{1}{2}.$$

#### 2.4.2. Post-Selection Shrinkage Estimation

We now propose a shrinkage estimator that combines information from two post-selection estimators, ${\hat{\beta }}^{\mathrm{RE}}$ and ${\hat{\beta }}^{\mathrm{WR}}$. Recall that

$${\hat{\beta }}_{{\hat{S}}_{1}}^{\mathrm{WR}}\;=\;{\left({\hat{\beta }}_{j}^{r},\;j\in {\hat{S}}_{1}\right)}^{T},\;\mathrm{and}\;{\hat{\beta }}_{{\hat{S}}_{2}}^{\mathrm{WR}}\;=\;{\left({\hat{\beta }}_{j}^{r}\;I\left(|{\hat{\beta }}_{j}^{r}|>a_{n}\right),\;j\in {\hat{S}}_{2}\right)}^{T}.$$

Define the post-selection shrinkage estimator for ${\hat{S}}_{1}$ as

(11) $${\hat{\beta }}_{{\hat{S}}_{1}}^{\mathrm{SE}}\;=\;{\hat{\beta }}_{{\hat{S}}_{1}}^{\mathrm{WR}}\;-\;\left(\frac{{\hat{s}}_{2}\;-\;2}{{\hat{T}}_{n}}\right)\left({\hat{\beta }}_{{\hat{S}}_{1}}^{\mathrm{WR}}\;-\;{\hat{\beta }}_{{\hat{S}}_{1}}^{\mathrm{RE}}\right),$$

where ${\hat{s}}_{2}=\left|{\hat{S}}_{2}\right|$, and ${\hat{\beta }}_{{\hat{S}}_{1}}^{\mathrm{RE}}$ is the restricted estimator obtained by maximizing the partial log-likelihood (3) over the set ${\hat{S}}_{1}$. The term ${\hat{T}}_{n}$ is given by

(12) $${\hat{T}}_{n}\;=\;{\left({\hat{\beta }}_{{\hat{S}}_{2}}^{\mathrm{WR}}\right)}^{T}{\left(X_{{\hat{S}}_{2}}^{T}\;M_{{\hat{S}}_{1}}\;X_{{\hat{S}}_{2}}\right)}^{-1}{\hat{\beta }}_{{\hat{S}}_{2}}^{\mathrm{WR}},\;M_{{\hat{S}}_{1}}\;=\;I_{n}\;-\;X_{{\hat{S}}_{1}}\;{\hat{\Sigma }}_{{\hat{S}}_{1}}^{-1}\;X_{{\hat{S}}_{1}}^{T},$$

using a generalized inverse if ${\hat{\Sigma }}_{{\hat{S}}_{1}}$ is singular.

To avoid over-shrinking when ${\hat{\beta }}_{{\hat{S}}_{1}}^{\mathrm{WR}}$ and ${\hat{\beta }}_{{\hat{S}}_{1}}^{\mathrm{SE}}$ have different signs, we define a *positive* shrinkage estimator via the convex combination

(13) $${\hat{\beta }}_{{\hat{S}}_{1}}^{\mathrm{PSE}}\;=\;{\hat{\beta }}_{{\hat{S}}_{1}}^{\mathrm{WR}}\;-\;\left(\left(\frac{{\hat{s}}_{2}-2}{{\hat{T}}_{n}}\right)\;\wedge \;1\right)\left({\hat{\beta }}_{{\hat{S}}_{1}}^{\mathrm{WR}}\;-\;{\hat{\beta }}_{{\hat{S}}_{1}}^{\mathrm{RE}}\right).$$

This modification is essential to prevent an overly aggressive shrinkage that might reverse the sign of estimates in ${\hat{\beta }}_{{\hat{S}}_{1}}^{\mathrm{WR}}$.

## 3. Asymptotic Properties

In this section, we study the asymptotic properties of the the post-selection shrinkage estimators for the Cox regression model. To investigate the asymptotic theory, we need the following regularity conditions to be met.

**(B1)**  $p=\exp(O\left(n^{\alpha }\right))$ for some 0<α<1.
**(B2)**  $\varrho_{1}=O\left(n^{-\eta }\right)$, where τ<η≤1 for τ in **(A2)**.
**(B3)**  The existence of a positive definite matrix $\Sigma_{n}$ such that $\lim_{n\to \infty }\Sigma_{n}=\Sigma$, where the eigenvalues of Σ satisfy $0<\kappa_{1}<\lambda_{\min}\left(\Sigma \right)\leq \lambda_{\max}\left(\Sigma \right)<\kappa_{2}<\infty$.
**(B4)**  Sparse Riesz condition: For the random design matrix X, any S⊂M with |S|=q,q≤p, and any vector $v\in R^{q}$, there exists $0<c_{*}<c^{*}<\infty$ such that $c_{*}\leq \left|\right|X_{S}^{T}{v||}_{2}^{2}/{\left||v|\right|}_{2}^{2}\leq c^{*}$ holds with probability tending to 1.

The following theorems will make it easier to compute the asympotic distributional bias (ADB) and asympotic distributional risk (ADR) of the proposed estimators:

**Theorem** **1.** *Suppose that assumptions (***A1***)–(***A3***) and* **(B1)***–***(B4)** *hold. If we choose $r_{n}=c_{2}a_{n}^{-2}{\left(\log\log n\right)}^{3}\log\left(n\vee p\right)$ for some constant $c_{2}>0$ and $a_{n}$ defined in (10) with ν<(η−α−τ)/3, then, ${\hat{S}}_{2}$ in (9) satisfies*

(14) $$\lim_{n\to \infty }P\left({\hat{S}}_{2}=S_{2}|{\hat{S}}_{1}=S_{1}\right)=1,$$

*where τ,η, and α are defined in (***A2***), (***B1***), and (***B2***), respectively.*

**Theorem** **2.** *Let $s_{n}^{2}=d_{n}^{T}\Sigma_{n}^{-1}d_{n}$ for any $(p_{1}+p_{2})\times 1$ vector $d_{n}$ satisfying $\left|\right|d_{n}{\left|\right|}_{2}^{2}\leq 1$. Suppose assumptions (* **B1** *)–(* **B4** *) hold. Consider a sparse Cox model with a signal strength under (* **A1** *)–(* **A3** *), and with 0<τ<1/2. Suppose a pre-selected model such as $S_{1}\subset {\hat{S}}_{1}\subset S_{1}\cup S_{2}$ is obtained with probability 1. If we choose $r_{n}$ in Theorem 1 with ν<{(η−α−τ)/,1/4−τ/2}, then, we have the asymptotic normality,*

(15) $$n^{1/2}s_{n}^{-1}d_{n}^{T}\left({\hat{\beta }}_{S_{\mathrm{null}}^{c}}^{WR}-\beta_{S_{\mathrm{null}}^{c}}\right)\overset{D}{⟶}\;N\left(0,1\right).$$

### Asymptotic Distributional Bias and Risk Analysis

In order to compare the estimators, we use the asymptotic distributional bias (ADB) and the asymptotic risk (ADR) expressions of the proposed estimators.

**Definition** **1.** *For any estimator $\beta_{1n}^{⋄}$ and $p_{1}$-dimensional vector $d_{1n}$, satisfying $\left|\right|d_{1n}{\left|\right|}_{2}^{2}\leq 1$, the ADB and ADR of $d_{1n}^{T}\beta_{1n}^{⋄}$, respectively, are defined as*

(16) $$\begin{array}{cc} ADB(d_{1n}^{T}\beta_{1n}^{⋄}) & =\lim_{n\to \infty }E\left[\left\{n^{1/2}s_{1n}^{-1}d_{1n}^{T}\left(\beta_{1n}^{⋄}-\beta_{1}\right)\right\}\right], \end{array}$$

(17) $$\begin{array}{cc} ADR(d_{1n}^{T}\beta_{1n}^{⋄}) & =\lim_{n\to \infty }E\left[{\left\{n^{1/2}s_{1n}^{-1}d_{1n}^{T}\left(\beta_{1n}^{⋄}-\beta_{1}\right)\right\}}^{2}\right], \end{array}$$

*where $s_{1n}^{2}=d_{1n}^{T}\Sigma_{S_{1}|S_{2}}^{-1}d_{1n}$. Let $\delta ={\left(\delta_{1},\cdots ,\delta_{p_{2}}\right)}^{T}\in R^{p_{2}}$ and*

(18) $$\Delta_{d_{1n}}=\frac{d_{1n}^{T}\left(\Sigma_{S_{1}}^{-1}\Sigma_{S_{1}S_{2}}\delta \delta^{T}\Sigma_{S_{2}S_{1}}\Sigma_{S_{1}}^{-1}\right)d_{1n}}{d_{1n}^{T}\left(\Sigma_{S_{1}}^{-1}\Sigma_{S_{1}S_{2}}\Sigma_{S_{2}|S_{1}}^{-1}\Sigma_{S_{2}S_{1}}\Sigma_{S_{1}}^{-1}\right)d_{1n}}.$$

*We have the following theorems on the expression of ADBs and ADRs of the post-selection estimators.*

**Theorem** **3.** *Let $d_{1n}$ be any $p_{1}$-dimensional vector satisfying $0<||d_{1n}{\left|\right|}_{2}^{2}\leq 1$ and $s_{1n}^{2}=d_{1n}^{T}\Sigma_{S_{1}|S_{2}}^{-1}d_{1n}$. Under the assumptions (* **A1)** *–(* **A3)** *, we have*

(19) $$\begin{array}{cc} ADB(d_{1n}^{T}{\hat{\beta }}_{1n}^{WR}) & =0, \end{array}$$

(20) $$\begin{array}{cc} ADB(d_{1n}^{T}{\hat{\beta }}_{1n}^{RE}) & =s_{1}^{-1}d_{2}^{T}\beta_{2}, \end{array}$$

(21) $$\begin{array}{cc} ADB(d_{1n}^{T}{\hat{\beta }}_{1n}^{SE}) & =\left(p_{2}-2\right)s_{1}^{-1}d_{2}^{T}\beta_{2}^{*}E\left[\chi_{p_{2}}^{-2}\left(\Delta_{d_{2}}\right)\right], \end{array}$$

(22) $$\begin{array}{cc} ADB(d_{1n}^{T}{\hat{\beta }}_{1n}^{PSE}) & =s_{1}^{-1}d_{2}^{T}\beta_{2}^{*}[\left(p_{2}-2\right)\left\{E\left[\chi_{p_{2}}^{-2}\left(\Delta_{d_{2}}\right)\right]+E\left[\chi_{p_{2}}^{-2}\left(\Delta_{d_{2}}\right)I\left(\chi_{p_{2}}^{2}\left(\Delta_{d_{2}}\right)<\left(p_{2}-2\right)\right)\right]\right\}\\ & -H_{p_{2}}\left(p_{2}-2;\Delta_{d_{2}}\right)], \end{array}$$

*where $d_{2n}=\Sigma_{S_{2}S_{1}}\Sigma_{S_{1}}^{-1}d_{1n}$ and $E\left[\chi_{p_{2}}^{-2j}\left(\Delta_{d_{2}}\right)\right]=\int_{0}^{\infty }x^{-2j}dH_{p_{2}}\left(x;\Delta_{d_{2}}\right)$.*

See the Appendix A for a detailed proof.

**Theorem** **4.** *Under the assumptions of Theorem 2, except (***A2)** *is replaced by $\beta_{j}=\delta /\sqrt{n}$, for $j\in S_{2}$, with $|\delta_{j}|<\delta_{\max}$, for some $\delta_{\max}>0$, we have*

(23) $$\begin{array}{cc} ADR(d_{1n}^{T}{\hat{\beta }}_{1n}^{WR}) & =1, \end{array}$$

(24) $$\begin{array}{cc} ADR(d_{1n}^{T}{\hat{\beta }}_{1n}^{RE}) & =1+{\left(1-c\right)}^{1/2}\left[2+{\left(1-c\right)}^{1/2}\left(1+2\Delta_{1}\right)\right], \end{array}$$

(25) $$\begin{array}{cc} ADR(d_{1n}^{T}{\hat{\beta }}_{1n}^{SE}) & =1+{\left(1-c\right)}^{1/2}\left(p_{2}-2\right)[{\left(1-c\right)}^{1/2}\left(p_{2}-2\right)\{E\left[\chi_{p_{2}+2}^{-4}\left(\Delta_{d_{2}}\right)\right]\\ & +{\left(s_{2}^{-1}d_{2}^{T}\beta_{2}\right)}^{2}E\left[\chi_{p_{2}}^{-4}\left(\Delta_{d_{2}}\right)\right]\}+2E\left[\chi_{p_{2}+2}^{-2}\left(\Delta_{d_{2}}\right)\right]], \end{array}$$

(26) $$\begin{array}{cc} ADR(d_{1n}^{T}{\hat{\beta }}_{1n}^{PSE}) & =1+\left(1-c\right){\left(p_{2}-2\right)}^{2}\{E\left[\chi_{p_{2}+2}^{-4}\left(\Delta_{d_{2}}\right)\right]+{\left(s_{2}^{-1}d_{2}^{T}\beta_{2}\right)}^{2}E\left[\chi_{p_{2}}^{-4}\left(\Delta_{d_{2}}\right)\right]\\ & +E[\chi_{p_{2}+2}^{-4}\left(\Delta_{d_{2}}\right)I\left(\chi_{p_{2}+2}^{2}\left(\Delta_{d_{2}}\right)<\left(p_{2n}-2\right)\right)]\}\\ & +2{\left(1-c\right)}^{1/2}\left(p_{2}-2\right)\{E\left[\chi_{p_{2}+2}^{-2}\left(\Delta_{d_{2}}\right)\right]+E\left[\chi_{p_{2}+2}^{-2}\left(\Delta_{d_{2}}\right)I\left(\chi_{p_{2}+2}^{2}\left(\Delta_{d_{2}}\right)<\left(p_{2}-2\right)\right)\right]\\ & -\left(p_{2}-2\right)E\left[\chi_{p_{2}+2}^{-4}\left(\Delta_{d_{2}}\right)I\left(\chi_{p_{2}+2}^{2}\left(\Delta_{d_{2}}\right)<\left(p_{2}-2\right)\right)\right]-{\left(1-c\right)}^{1/2}\\ & \times [E[\chi_{p_{2}+2}^{-2}\left(\Delta_{d_{2}}\right)I\left(\chi_{p_{2}+2}^{2}\left(\Delta_{d_{2}}\right)<\left(p_{2}-2\right)\right)]\\ & +{\left(s_{2}^{-1}d_{2}^{T}\beta_{2}^{*}\right)}^{2}E\left[\chi_{p_{2}}^{-2}\left(\Delta_{d_{2}}\right)I\left(\chi_{p_{2}}^{2}\left(\Delta_{d_{2}}\right)<\left(p_{2}-2\right)\right)\right]]\},\\ & +{\left(1-c\right)}^{1/2}[{\left(1-c\right)}^{1/2}\left(E\left[\chi_{p_{2}+2}^{2}\left(\Delta_{d_{2}}\right)\right]+{\left(s_{2}^{-1}d_{2}^{T}\beta_{2}^{*}\right)}^{2}H_{p_{2}}\left(p_{2}-2;\Delta_{d_{2}}\right)\right)\\ & +2\left\{H_{p_{2}}\left(p_{2}-2;\Delta_{d_{2}}\right)-\left(p_{2}-2\right)E\left[\chi_{p_{2}+2}^{-2}\left(\Delta_{d_{2}}\right)I\left(\chi_{p_{2}+2}^{2}\left(\Delta_{d_{2}}\right)<\left(p_{2}-2\right)\right)\right]\right\}], \end{array}$$

*where $c=\lim_{n\to \infty }d_{1n}^{T}\Sigma_{S_{1}}^{-1}d_{1n}/\left(d_{1n}^{T}\Sigma_{S_{11.2}}^{-1}d_{1n}\right)\leq 1$ and $s_{2n}^{2}=d_{2n}^{T}\Sigma_{S_{22.1}}^{-1}d_{2n}$.*

It can be observed that the theoretical results are different from Theorem 3 of [19]. Ref. [19] considered the ADR of PSE estimations for the linear model. In contrast, our Theorems 3 and 4 are used for the PSE with the Cox proportional hazards model, which are feasible estimations. From Theorem 4, we can compare the ADRs of the estimators.

**Corollary** **1.** *Under the assumptions in Theorem 4, we have*

1. *If ${\left||\delta |\right|}_{2}^{2}\leq 1$, then $ADR\left(d_{1n}^{T}{\hat{\beta }}_{1n}^{PSE}\right)\leq ADR\left(d_{1n}^{T}{\hat{\beta }}_{1n}^{SE}\right)\leq ADR\left(d_{1n}^{T}{\hat{\beta }}_{1n}^{WR}\right)$;*
2. *If ${\left||\delta |\right|}_{2}^{2}=o\left(1\right)$ and $p_{2}\to \infty$, then $ADR\left(d_{1n}^{T}{\hat{\beta }}_{1n}^{RE}\right)<ADR\left(d_{1n}^{T}{\hat{\beta }}_{1n}^{PSE}\right)\leq ADR\left(d_{1n}^{T}{\hat{\beta }}_{1n}^{WR}\right)$ for δ=0.*

Corollary 1 shows that the performance of the post-selection PSE is closely related to the RE. On the ond hand, if ${\hat{s}}_{1}\subset S_{1}\cup S_{2}$ and $\left(S_{1}\cup S_{2}\right)\cap {\hat{S}}_{1}^{c}$ are large, then the post-selection PSE tends to dominate the RE. Further, if a variable selection method generates the right submodel and ${\left||\delta |\right|}_{2}^{2}=o\left(1\right)$, that is, $\lim_{n\to \infty }{\hat{S}}_{1}=S_{1}\cup S_{2}$, then, a post-selection likelihood estimator ${\hat{\beta }}_{1n}^{RE}$ is the most efficient one compared with all other post-selection estimators.

**Remark** **1.** *The simultaneous variable selection and parameter estimation may not lead to a good estimation strategy when weak signals co-exist with zero signals. Even though the selected candidate subset models can be provided by some existing variable selection techniques when p>n, the prediction performance can be improved by the post-selection shrinkage strategy, especially when an under-fitted subset model is selected by an aggressive variable selection procedure.*

## 4. Simulation Study

In this section, we present a simulation study designed to compare the quadratic risk performance of the proposed estimators under the Cox regression model. Each row of the design matrix X is generated i.i.d. from a N(0,Σ) distribution, where Σ follows an autoregressive covariance structure, as follows:

$$\Sigma_{jj^{\prime }}\;=\;0.5^{\;|j-j^{\prime }|},\;1\;\leq \;j,j^{\prime }\;\leq \;p.$$

In this setup, we consider the following true regression coefficients:

(27) $$\beta \;=\;{\left(\underset{S_{1}}{\underset{︸}{8,\;9,\;10}},\;\underset{S_{2}}{\underset{︸}{1,\;0.8,\;0.5,\;\underset{p_{2}-p_{1}}{\underset{︸}{0.2,\ldots ,0.2}}}},\;\underset{p-p_{1}-p_{2}}{\underset{︸}{0,\;0,\;0,\;\ldots ,\;0}}\right)}^{T},$$

where the subsets $S_{1}$ and $S_{2}$ correspond to strong and weak signals, respectively. The true survival times Y are generated from an exponential distribution with parameter Xβ. Censoring times are drawn from a Uniform(0,c) distribution, where *c* is chosen to achieve the desired censoring rate. We consider censoring rates of 15% and 25%, and we explore sample sizes n=100,300,400.

We compare the performance of our proposed estimators against two well-known penalized likelihood methods, namely, LASSO and Elastic Net (ENet). We employ the R package glmnet to fit these penalized methods and choose the tuning parameters via cross-validation. For each combination of *n* and *p*, we run 1000 Monte Carlo simulations. Let $\beta_{1n}^{⋄}$ denote either ${\hat{\beta }}_{1n}^{\mathrm{PSE}}$ or ${\hat{\beta }}_{1n}^{\mathrm{RE}}$ after variable selection. We assess the performance using the relative mean squared error (RMSE) with respect to ${\hat{\beta }}_{1n}^{\mathrm{WR}}$ as follows:

$$\mathrm{RMSE}\;\left(\beta_{1n}^{⋄}\right)\;=\;\frac{E\;{∥{\hat{\beta }}_{1n}^{\mathrm{WR}}-\beta ∥}_{2}^{2}}{E\;{∥\beta_{1n}^{⋄}-\beta ∥}_{2}^{2}}.$$

An $\mathrm{RMSE}(\beta_{1n}^{⋄})>1$ indicates that $\beta_{1n}^{⋄}$ outperforms ${\hat{\beta }}_{1n}^{\mathrm{WR}}$, and a larger RMSE signifies a stronger degree of superiority over ${\hat{\beta }}_{1n}^{\mathrm{WR}}$.

Table 1 presents the relative mean squared error (RMSE) values for different regression methods—LASSO and Elastic Net (ENet)—under varying sample sizes (*n*), number of predictors (*p*), and censoring percentages (15% and 25%). The RMSE values are averaged over 1000 simulation runs. The table compares three estimators, ${\hat{\beta }}_{S_{1}}^{PLE}$, ${\hat{\beta }}_{S_{1}}^{RE}$, and ${\hat{\beta }}_{S_{1}}^{PSE}$, providing insight into their performance under different settings.

**Figure 1:**
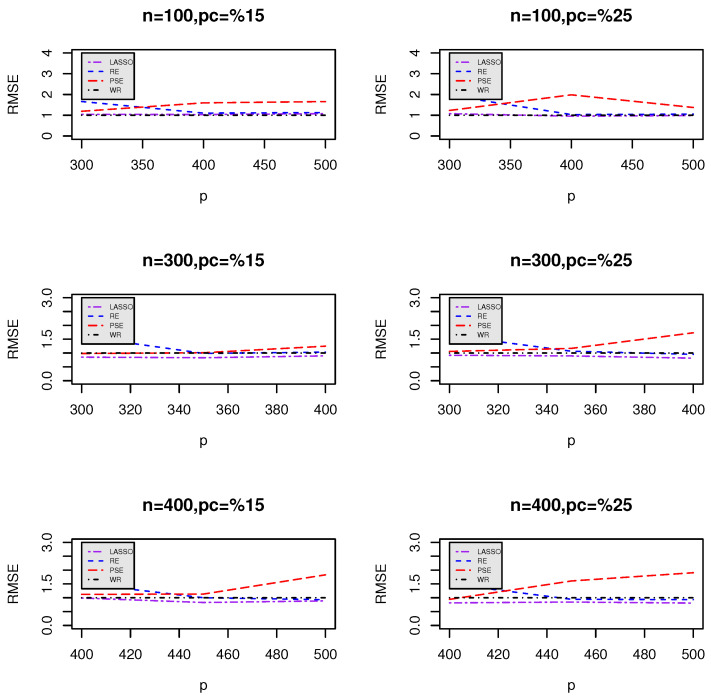
Relative mean squared error (RMSE) of the proposed estimators compared to LASSO for different *n* and *p*.

**Figure 2:**
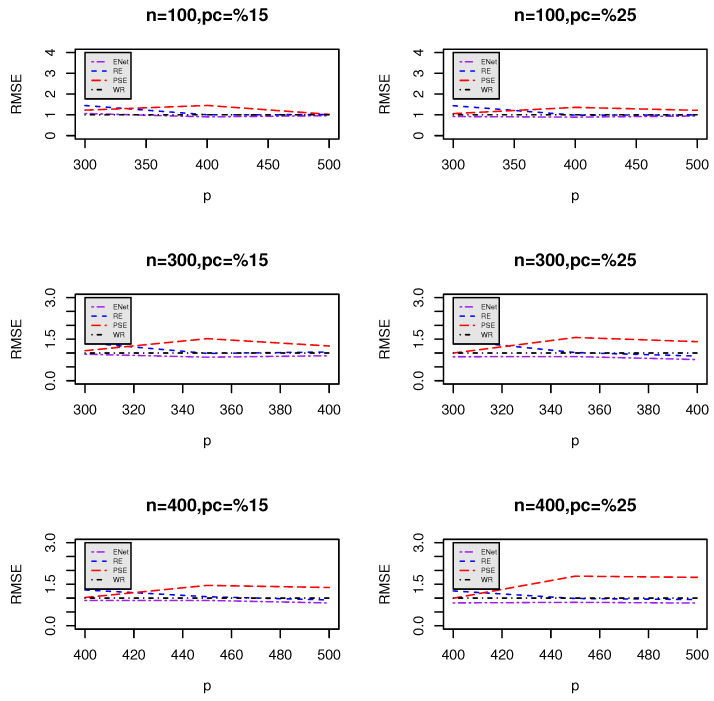
Relative mean squared error (RMSE) of the proposed estimators compared to Elastic Net for different *n* and *p*.

Figure 1 and Figure 2 visualize the RMSE trends for different values of *p* when comparing LASSO (Figure 1) and ENet (Figure 2) against the proposed estimators (RE and PSE). The plots indicate how RMSE varies as *p* increases for different sample sizes (*n*) and censoring levels.

### Key Observations and Insights

1. **Superior performance of post-selection estimators:** Across all combinations of *n* and *p*, the post-selection estimators (${\hat{\beta }}_{S_{1}}^{RE}$ and ${\hat{\beta }}_{S_{1}}^{PSE}$) consistently demonstrate lower RMSEs compared to LASSO and ENet. This suggests that these estimators provide better predictive accuracy and stability.
2. **Impact of censoring percentage:**
  - When the censoring percentage increases from 15% to 25%, the RMSE values tend to increase across all methods, indicating the expected loss of predictive power due to increased censoring.
  - However, the post-selection estimators maintain a more stable RMSE trend, demonstrating their robustness in handling censored data.
3. **Effect of increasing predictors (*p*):**
  - As *p* increases, the RMSE for LASSO and ENet tends to rise, particularly under higher censoring rates.
  - This trend suggests that LASSO and ENet struggle with larger feature spaces, likely due to their tendency to aggressively shrink weaker covariates.
  - In contrast, the post-selection estimators show relatively stable RMSE behavior, indicating their ability to retain relevant information even in high-dimensional settings.
4. **Impact of sample size (*n*) on RMSE stability:**
  - Larger sample sizes (*n*) generally lead to lower RMSE values across all methods.
  - However, the gap between LASSO/ENet and the post-selection estimators remains consistent, reinforcing the advantage of the proposed methods even with more data.
5. **Comparing LASSO and ENet:**
  - ENet generally has lower RMSE values than LASSO, particularly for small sample sizes, indicating its advantage in balancing feature selection and regularization.
  - However, ENet still underperforms compared to post-selection estimators, suggesting that the additional shrinkage adjustments help mitigate underfitting issues.

To further compare the sparsity of the coefficient estimators, we also measure the False Positive Rate (FPR), as follows:

(28) $$\begin{array}{c} \mathrm{FPR}\;\left(\hat{\beta }\right)\;=\;\frac{\;\left|\left\{\;j:{\hat{\beta }}_{j}\neq 0\;\wedge \;\beta_{j}=0\right\}\right|}{\;\left|\left\{\;j:\beta_{j}=0\right\}\right|}\;. \end{array}$$

A higher FPR indicates that more non-informative variables are incorrectly included in the model, thereby complicating interpretation [22]. When β does not contain any zero components, the FPR is undefined. Table 2 compares the performance of LASSO and Elastic Net (ENet) in selecting variables in a high-dimensional Cox model under 15% and 25% censoring. As sample size (*n*) increases, both methods select more variables, but false positive rates (FPR) also rise, especially for ENet. LASSO is more conservative, selecting fewer variables with a lower FPR, while ENet selects more but at the cost of higher false discoveries. Higher censoring (25%) slightly increases FPR, reducing selection accuracy. Overall, LASSO offers better false positive control, whereas ENet captures more variables but with increased risk of selecting irrelevant ones.

## 5. Real Data Example

In this section, we illustrate the practical utility of our proposed methodology on two different high-dimensional datasets.

### 5.1. Example 1

We first apply our method to a gene expression dataset comprising n=614 breast cancer patients, each with p=1490 genes. All patients received anthracycline-based chemotherapy. Among these 614 individuals, there were 134 (21%) censored observations, and the mean time to treatment response was approximately 2.98 years. Using biological pathways to identify important genes, Ref. [23] previously selected 29 genes and reported a maximum area under the receiver operating characteristic curve (AUC) of about 62%. This relatively low AUC suggests limited predictive power when only using these 29 genes.

To improve upon these findings, we begin by performing an initial noise-reduction procedure on the data. This step helps remove potential outliers and irrelevant features, thereby enhancing the quality of the subsequent variable selection and estimation processes. We applied LASSO and Elastic Net (ENet) for gene selection. The results show that LASSO selected 14 genes, whereas ENet selected 12 genes. We then applied the proposed post-selection shrinkage estimators introduced in Section 2 to evaluate their performance compared to standard methods such as LASSO and Elastic Net. Table 3 shows the estimated coefficients from different estimators, along with the AUC at the bottom. It is evident that the PSE estimate has slightly improved the prediction performance.

### 5.2. Example 2

We now consider the diffuse large B-cell lymphoma (DLBCL) dataset of [24], which is also high-dimensional. This dataset was used as a primary example to illustrate the effectiveness of our proposed dimension-reduction method. It consists of measurements on 7399 genes obtained from 240 patients via customized cDNA microarrays (lymphochip). Each patient’s survival time was recorded, ranging from 0 to 21.8 years; 127 patients had died (uncensored) and 95 were alive (censored) at the end of the study. Additional details on the dataset can be found in [24].

To obtain the post-selection shrinkage estimators, we first selected candidate subsets using two variable selection approaches—LASSO and Elastic Net (ENet). All tuning parameters were chosen via 10-fold cross-validation. Table 4 shows the estimated coefficients from both LASSO and ENet for the setting p=6800. The AUC results indicate that ${\hat{\beta }}_{{\hat{S}}_{1}}^{\mathrm{PSE}}$ generally outperforms ${\hat{\beta }}_{{\hat{S}}_{1}}^{\mathrm{RE}}$ and ${\hat{\beta }}_{{\hat{S}}_{1}}^{\mathrm{PLE}}$ for both LASSO and ENet procedures. Notably, the ENet-based estimators appear more robust than those obtained via LASSO, underscoring the value of combining $L_{1}$ and $L_{2}$ penalties in high-dimensional survival analysis.

## 6. Conclusions

In this paper, we proposed high-dimensional post-selection shrinkage estimators for Cox’s proportional hazards models based on the work of [19]. We investigated the asymptotic risk properties of these estimators in relation to the risks of the subset candidate model, as well as the LASSO and ENet estimators. Our results indicate that the new estimators perform particularly well when the true model contains weak signals. The proposed strategy is also conceptually intuitive and computationally straightforward to implement.

Our theoretical analysis and simulation studies demonstrate that the post-selection shrinkage estimator exhibits superior performance relative to LASSO and ENet, in part because it mitigates the loss of efficiency often associated with variable selection. As a powerful tool for producing interpretable models, sparse modeling via penalized regularization has become increasingly popular for high-dimensional data analysis. Our post-selection shrinkage estimator preserves model interpretability while enhancing predictive accuracy compared to existing penalized regression techniques. Furthermore, two real-data examples illustrate the practical advantages of our method, confirming that its performance is robust and potentially valuable for a range of high-dimensional applications.

## Figures and Tables

**Table 1:** Simulated relative mean squared error (RMSE) across different values of *p* and *n*, averaged over N=1000 simulation runs.

			Censoring Percentage					
			**15%**			**25%**		
n	p	**Method**	${\hat{\beta }}_{{\hat{S}}_{1}}^{\mathrm{PLE}}$	${\hat{\beta }}_{{\hat{S}}_{1}}^{\mathrm{RE}}$	${\hat{\beta }}_{{\hat{S}}_{1}}^{\mathrm{PSE}}$	${\hat{\beta }}_{{\hat{S}}_{1}}^{\mathrm{PLE}}$	${\hat{\beta }}_{{\hat{S}}_{1}}^{\mathrm{RE}}$	${\hat{\beta }}_{{\hat{S}}_{1}}^{\mathrm{PSE}}$
100	300	LASSO	1.04	1.66	1.19	1.08	1.96	1.23
		ENet	1.07	1.45	1.23	0.92	1.44	1.06
	400	LASSO	1.03	1.10	1.60	0.96	1.03	1.98
		ENet	0.90	1.00	1.45	0.89	0.98	1.36
	500	LASSO	1.08	1.13	1.66	0.98	1.05	1.37
		ENet	0.96	1.01	1.03	0.95	1.00	1.22
300	300	LASSO	0.85	1.60	0.98	0.92	1.64	1.06
		ENet	0.96	1.37	1.08	0.87	1.46	1.00
	350	LASSO	0.83	0.99	1.01	0.90	1.07	1.17
		ENet	0.85	0.99	1.52	0.87	1.02	1.56
	400	LASSO	0.90	1.03	1.25	0.81	0.95	1.73
		ENet	0.90	1.04	1.25	0.76	0.89	1.41
400	400	LASSO	0.99	1.52	1.12	0.82	1.50	0.94
		ENet	0.91	1.29	1.02	0.83	1.26	0.99
	450	LASSO	0.83	1.00	1.13	0.84	0.94	1.61
		ENet	0.92	1.05	1.46	0.85	0.99	1.79
	500	LASSO	0.89	0.93	1.83	0.81	0.93	1.90
		ENet	0.82	0.93	1.38	0.82	0.95	1.75

**Table 2:** Average number of selected predictors (${\hat{S}}_{1}$) and false positive rate (FPR) across different values of *n* and *p*, averaged over N=1000 simulation runs.

			Censoring Percentage			
			%15		%25	
n	p	**Method**	**Average** ${\hat{S}}_{1}$	**FPR**	**Average** ${\hat{S}}_{1}$	**FPR**
100	300	LASSO	6.1	0.063	6.4	0.056
		ENet	6.2	0.063	6.6	0.052
	400	LASSO	4.9	0.072	5.2	0.085
		ENet	5.1	0.072	4.8	0.075
	500	LASSO	5.6	0.039	12.6	0.043
		ENet	4.9	0.039	4.0	0.033
300	300	LASSO	13.4	0.209	13.8	0.223
		ENet	12.9	0.209	16.3	0.282
	350	LASSO	15.6	0.202	15.8	0.208
		ENet	15.7	0.202	22.6	0.279
	400	LASSO	14.5	0.137	13.7	0.155
		ENet	13.5	0.137	14.2	0.173
400	400	LASSO	14.1	0.163	15.8	0.171
		ENet	14.2	0.163	20.4	0.212
	450	LASSO	18.4	0.217	23.5	0.24
		ENet	19.1	0.217	30.1	0.263
	500	LASSO	13.6	0.150	13.3	0.158
		ENet	13.3	0.150	13.6	0.158

**Table 3:** Estimated coefficients using the LASSO and ENet method for example 1.

	LASSO			ENet		
**Gen ID**	${\hat{\beta }}_{{\hat{S}}_{1}}^{\mathrm{LASSO}}$	${\hat{\beta }}_{{\hat{S}}_{1}}^{\mathrm{RE}}$	${\hat{\beta }}_{{\hat{S}}_{1}}^{\mathrm{PSE}}$	${\hat{\beta }}_{{\hat{S}}_{1}}^{\mathrm{ENet}}$	${\hat{\beta }}_{{\hat{S}}_{1}}^{\mathrm{RE}}$	${\hat{\beta }}_{{\hat{S}}_{1}}^{\mathrm{PSE}}$
18	−0.02	0.26	0.21	−0.03	0.07	0.20
97	0.01	0.27	0.00	0.01	0.26	0.01
101	0.05	0.19	0.13	0.05	0.27	0.12
128	–	–	–	−0.01	–	–
232	0.04	−0.42	−0.28	0.04	0.20	−0.25
342	0.15	−0.42	−0.13	0.14	−0.39	−0.10
369	−0.09	−0.05	0.04	−0.08	−0.40	−0.12
408	−0.01	–	–	−0.01	−0.09	0.03
410	0.03	−0.26	−0.15	0.03	−0.06	−0.14
445	–	–	–	−0.00	–	–
468	0.14	0.08	0.02	0.13	−0.26	−0.01
660	−0.00	–	–	−0.00	–	–
731	−0.08	0.09	0.06	−0.08	0.06	0.06
810	−0.04	−0.08	−0.09	0.01	0.09	−0.09
907	–	–	–	0.01	–	–
934	−0.00	–	–	−0.00	–	–
952	–	–	–	−0.01	–	–
961	−0.05	—	–	−0.05	−0.08	0.20
1212	–	–	–	−0.00	–	–
AUC	0.62	0.63	0.65	0.63	0.64	0.66

**Table 4:** Estimated coefficients using the LASSO and ENet method for example 2.

	LASSO			ENet		
**Gen ID**	${\hat{\beta }}_{{\hat{S}}_{1}}^{\mathrm{LASSO}}$	${\hat{\beta }}_{{\hat{S}}_{1}}^{\mathrm{RE}}$	${\hat{\beta }}_{{\hat{S}}_{1}}^{\mathrm{PSE}}$	${\hat{\beta }}_{{\hat{S}}_{1}}^{\mathrm{ENet}}$	${\hat{\beta }}_{{\hat{S}}_{1}}^{\mathrm{RE}}$	${\hat{\beta }}_{{\hat{S}}_{1}}^{\mathrm{PSE}}$
95	0.02	–	–	−0.34	–	–
112	0.06	0.71	0.70	−0.00	−0.13	−0.08
173	−0.63	–	–	0.68	–	–
205	–	–	–	–	–	–
551	1.60	1.69	1.57	−0.11	−0.28	−0.20
1377	−0.22	−0.84	−0.80	−0.09	−0.16	−0.12
1526	0.41	0.67	0.56	0.02	–	–
1543	−0.43	−0.79	−0.77	0.40	0.75	0.69
2003	−0.11	–	–	1.10	–	–
2025	0.18	0.90	0.78	1.04	1.22	1.07
2439	–	–	–	−0.01	−0.14	−0.12
2705	−0.85	–	–	0.36	0.77	0.61
2973	0.59	1.23	0.99	−0.63	−1.12	−0.81
3240	1.13	–	–	0.03	–	–
3598	−0.22	−0.59	−0.54	0.29	0.55	0.49
3882	0.13	0.40	0.39	−0.06	−0.20	−0.15
4015	0.34	0.81	0.76	−0.08	−0.13	−0.12
4186	−0.50	−0.72	−0.53	–	–	–
4357	0.09	–	–	−0.59	−0.70	−0.65
4662	0.70	0.90	0.83	0.21	0.60	0.38
5131	0.54	0.80	0.71	0.01	0.01	0.01
5222	−0.15	−0.38	−0.26	1.24	1.67	1.34
5541	–	–	–	−0.52	−0.72	−0.68
5577	0.39	0.86	0.70	−0.73	−0.97	−0.80
5778	−0.62	–	–	−0.09	–	–
5808	–	–	–	0.35	0.55	0.46
5951	–	–	–	1.29	2.12	1.70
6103	–	–	–	−0.63	−0.80	−0.76
6254	–	–	–	0.25	0.56	0.48
6493	–	–	–	0.65	–	–
6510	–	–	–	0.86	1.09	0.99
AUC	0.71	0.71	0.73	0.72	0.72	0.74

## Data Availability

All data that are used in this study is publicly available.

## Nomenclature

**Symbol**	**Description**
**General Notation**	
*n*	Sample size (number of observations)
*p*	Number of covariates (predictor variables)
R	Set of real numbers
P	Probability measure
E	Expectation operator
I(·)	Indicator function
**Regression and Estimators**	
β	Regression coefficient vector
$\hat{\beta }$	Estimated regression coefficients
λ	Regularization parameter (for LASSO/ENet)
${\hat{S}}_{1}$	Selected subset of variables
$d_{1n}$	$p_{1}$-dimensional vector in the selection model
$\beta_{1n}^{\circ }$	Selected regression coefficient estimator
${\hat{\beta }}_{WR}$	Weighted Ridge (WR) estimator
**Survival Analysis Notation**	
L(β)	Cox proportional hazards likelihood function
D	Dataset containing observations
*X*	Covariate matrix
*Y*	Response variable (time-to-event outcome)
h(t)	Hazard function at time *t*
$\hat{h}\left(t\right)$	Estimated hazard function
Λ(t)	Cumulative hazard function
**Evaluation Metrics**	
RMSE	Root Mean Squared Error
FPR	False Positive Rate
AUC	Area Under the Curve (for classification models)
**Methods and Models**	
LASSO	Least Absolute Shrinkage and Selection Operator
ENet	Elastic Net
Cox-PH	Cox Proportional Hazards Model
WR	Weighted Ridge estimator
PSE	Post-selection Shrinkage Estimator
RE	Restricted Estimator

## Appendix A. Proofs

The technical proofs of Theorems 3 and 4 are included in this section.

**(Proof of Theorem 3).** Here, we provide the proof of the ADB expressions of the proposed estimators. Based on Theorem 2, it is clear that

$$\lim_{n\to \infty }E\left[n^{1/2}s_{1n}^{-1}d_{1n}^{T}\left({\hat{\beta }}_{1n}^{WR}-\beta_{1}\right)\right]=E\left[\lim_{n\to \infty }\left\{n^{1/2}s_{1n}^{-1}d_{1n}^{T}\left({\hat{\beta }}_{1n}^{WR}-\beta_{1}\right)\right\}\right]=E\left[Z\right]=0,$$

where Z∼N(0,1). Then,

$$\begin{array}{cc} ADB(d_{1n}^{T}{\hat{\beta }}_{1n}^{RE}) & =\lim_{n\to \infty }E\left[n^{1/2}s_{1n}^{-1}d_{1n}^{T}\left({\hat{\beta }}_{1n}^{RE}-\beta_{1}\right)\right]\\ & =\lim_{n\to \infty }E\left[n^{1/2}s_{1n}^{-1}d_{1n}^{T}\left\{\left({\hat{\beta }}_{1n}^{WR}-\beta_{1}\right)-\left({\hat{\beta }}_{1n}^{WR}-{\hat{\beta }}_{1n}^{RE}\right)\right\}\right]\\ & =\lim_{n\to \infty }E\left[n^{1/2}s_{1n}^{-1}d_{1n}^{T}\left({\hat{\beta }}_{1n}^{WR}-\beta_{1}\right)\right]-\lim_{n\to \infty }E\left[n^{1/2}s_{1n}^{-1}d_{1n}^{T}\left({\hat{\beta }}_{1n}^{WR}-{\hat{\beta }}_{1n}^{RE}\right)\right]\\ & =ADB\left(d_{1n}^{T}{\hat{\beta }}_{1n}^{WR}\right)-\lim_{n\to \infty }E\left[n^{1/2}s_{1n}^{-1}d_{1n}^{T}\left({\hat{\beta }}_{1n}^{WR}-{\hat{\beta }}_{1n}^{RE}\right)\right]\\ & =\lim_{n\to \infty }E\left[n^{1/2}s_{1n}^{-1}d_{2n}^{T}{\hat{\beta }}_{2n}^{WR}\right]\\ & =\lim_{n\to \infty }\left(s_{2n}/s_{1n}\right)E\left[n^{1/2}s_{2n}^{-1}d_{2n}^{T}{\hat{\beta }}_{2n}^{WR}\right]\\ \; & =\left(s_{2}/s_{1}\right)s_{2}^{-1}d_{2}^{T}\beta_{2}=s_{1}^{-1}d_{2}^{T}\beta_{2} \end{array}$$

where $d_{2n}=\Sigma_{S_{2}S_{1}}\Sigma_{S_{1}}^{-1}d_{1n}$, $d_{1n}^{T}\left({\hat{\beta }}_{1n}^{WR}-{\hat{\beta }}_{1n}^{RE}\right)=-d_{1}^{T}\Sigma_{S_{1}}^{-1}\Sigma_{S_{2}S_{1}}{\hat{\beta }}_{2n}^{WR}=-d_{2n}^{T}{\hat{\beta }}_{2n}^{WR}$ and $s_{2n}^{2}=d_{2}^{T}\Sigma_{S_{2}|S_{1}}^{-1}d_{2n}$. Now, we compute the ADB of ${\hat{\beta }}_{1n}^{SE}$ as follows

$$\begin{array}{cc} ADB(d_{1n}^{T}{\hat{\beta }}_{1n}^{SE}) & =\lim_{n\to \infty }E\left[n^{1/2}s_{1n}^{-1}d_{1n}^{T}\left({\hat{\beta }}_{1n}^{SE}-\beta_{1}\right)\right]\\ & =\lim_{n\to \infty }E\left[n^{1/2}s_{1n}^{-1}d_{1n}^{T}\left({\hat{\beta }}_{1n}^{WR}-\left[\left(p_{2n}-2\right){\hat{T}}_{n}^{-1}\right]\left({\hat{\beta }}_{1n}^{WR}-{\hat{\beta }}_{1n}^{RE}\right)-\beta_{1}\right)\right]\\ \; & =\lim_{n\to \infty }E\left[n^{1/2}s_{1n}^{-1}d_{1n}^{T}\left({\hat{\beta }}_{1n}^{WR}-\beta_{1}\right)\right]\\ & -\lim_{n\to \infty }\left(p_{2n}-2\right)E\left[n^{1/2}s_{1n}^{-1}d_{1n}^{T}\left({\hat{\beta }}_{1n}^{WR}-{\hat{\beta }}_{1}^{RE}\right){\hat{T}}_{n}^{-1}\right]\\ & =E\left[Z\right]-\left(p_{2}-2\right)E\left[\lim_{n\to \infty }\left\{n^{1/2}s_{1n}^{-1}d_{1n}^{T}\left({\hat{\beta }}_{1n}^{WR}-{\hat{\beta }}_{1n}^{RE}\right)T_{n}^{-1}\right\}\right]\\ & =\left(p_{2}-2\right)E\left[\lim_{n\to \infty }\left\{n^{1/2}s_{1n}^{-1}d_{2n}^{T}{\hat{\beta }}_{2n}^{WR}T_{n}^{-1}\right\}\right]\\ & =\left(p_{2}-2\right)\left(s_{2}/s_{1}\right)E\left[\lim_{n\to \infty }\left\{n^{1/2}s_{2n}^{-1}d_{2n}^{T}{\hat{\beta }}_{2n}^{WR}T_{n}^{-1}\right\}\right]\\ & =\left(p_{2}-2\right)s_{1}^{-1}d_{2}^{T}\beta_{2}E\left[\chi_{p_{2}}^{-2}\left(\Delta_{d_{2}}\right)\right]. \end{array}$$

Finally, we obtain the ADB of ${\hat{\beta }}_{1n}^{PSE}$,

$$\begin{array}{cc} ADB(d_{1n}^{T}{\hat{\beta }}_{1n}^{PSE}) & =\lim_{n\to \infty }E\left[n^{1/2}s_{1n}^{-1}d_{1n}^{T}\left({\hat{\beta }}_{1n}^{PSE}-\beta_{1}\right)\right]\\ & =\lim_{n\to \infty }E[n^{1/2}s_{1n}^{-1}d_{1n}^{T}\{{\hat{\beta }}_{1n}^{SE}+\left[1-\left(p_{2n}-2\right){\hat{T}}_{n}^{-1}\right]\left({\hat{\beta }}_{1n}^{WR}-{\hat{\beta }}_{1n}^{RE}\right)\\ & \;\;\;\times I\left({\hat{T}}_{n}<\left(p_{2n}-2\right)\right)-\beta_{1}\}]\\ & =\lim_{n\to \infty }E\left[n^{1/2}s_{1n}^{-1}d_{1n}^{T}\left({\hat{\beta }}_{1n}^{SE}-\beta_{1}\right)\right]\\ & +\lim_{n\to \infty }E\left[n^{1/2}s_{1n}^{-1}d_{1n}^{T}\left[1-\left(p_{2n}-2\right){\hat{T}}_{n}^{-1}\right]\left({\hat{\beta }}_{1n}^{WR}-{\hat{\beta }}_{1n}^{RE}\right)I\left({\hat{T}}_{n}<\left(p_{2n}-2\right)\right)\right]\\ & +E\left[\lim_{n\to \infty }\left\{n^{1/2}s_{1n}^{-1}d_{1n}^{T}\left({\hat{\beta }}_{1n}^{SE}-\beta_{1}\right)\right\}\right]\\ \; & +E\left[\lim_{n\to \infty }\left\{n^{1/2}s_{1n}^{-1}d_{1n}^{T}\left({\hat{\beta }}_{1n}^{WR}-{\hat{\beta }}_{1n}^{RE}\right)I\left({\hat{T}}_{n}<\left(p_{2n}-2\right)\right)\right\}\right]\\ & -\left(p_{2}-2\right)E\left[\lim_{n\to \infty }\left\{n^{1/2}s_{1n}^{-1}d_{1n}^{T}\left({\hat{\beta }}_{1n}^{WR}-{\hat{\beta }}_{1n}^{RE}\right)T_{n}^{-1}I\left({\hat{T}}_{n}<(p_{2n}-2\right))\right\}\right]\\ & =ADB\left(d_{1n}^{T}{\hat{\beta }}_{1n}^{SE}\right)-E\left[\lim_{n\to \infty }\left\{n^{1/2}s_{1n}^{-1}d_{2n}^{T}{\hat{\beta }}_{2n}^{WR}I\left({\hat{T}}_{n}<\left(p_{2n}-2\right)\right)\right\}\right]\\ & +\left(p_{2}-2\right)E\left[\lim_{n\to \infty }\left\{n^{1/2}s_{1n}^{-1}d_{2n}^{T}{\hat{\beta }}_{2n}^{WR}{\hat{T}}_{n}^{-1}I\left({\hat{T}}_{n}<\left(p_{2n}-2\right)\right)\right\}\right]\\ & =ADB\left(d_{1n}^{T}{\hat{\beta }}_{1n}^{SE}\right)-\left(s_{2}/s_{1}\right)E\left[ZI\left(\chi_{P_{2}}^{2}\left(\Delta_{d_{2}}\right)<\left(p_{2}-2\right)\right)\right]\\ & -s_{1}^{-1}d_{2}^{T}\beta_{2}H_{p_{2}}\left(p_{2}-2;\Delta_{d_{2}}\right)\\ & +\left(p_{2}-2\right)\left(s_{2}/s_{1}\right)E\left[Z\chi_{p_{2}}^{-2}\left(\Delta_{d_{2}}\right)I\left(\chi_{P_{2}}^{2}\left(\Delta_{d_{2}}\right)<\left(p_{2}-2\right)\right)\right]\\ & +\left(p_{2}-2\right)s_{1}^{-1}d_{2}^{T}\beta_{2}E\left[\chi_{p_{2}}^{-2}\left(\Delta_{d_{2}}\right)I\left(\chi_{P_{2}}^{2}\left(\Delta_{d_{2}}\right)<\left(p_{2}-2\right)\right)\right]\\ & =ADB\left(d_{1n}^{T}{\hat{\beta }}_{1n}^{SE}\right)-s_{1}^{-1}d_{2}^{T}\beta_{2}H_{p_{2}}\left(p_{2}-2;\Delta_{d_{2}}\right)\\ & +\left(p_{2}-2\right)s_{1}^{-1}d_{2}^{T}\beta_{2}E\left[\chi_{p_{2}}^{-2}\left(\Delta_{d_{2}}\right)I\left(\chi_{p_{2}}^{2}\left(\Delta_{d_{2}}\right)<\left(p_{2}-2\right)\right)\right]\\ & =s_{1}^{-1}d_{2}^{T}\beta_{2}[\left(p_{2}-2\right)\{E\left[\chi_{p_{2}}^{-2}\left(\Delta_{d_{2}}\right)]+E\left[\chi_{p_{2}}^{-2}\left(\Delta_{d_{2}}\right)I\left(\chi_{P_{2}}^{2}\left(\Delta_{d_{2}}\right)<\left(p_{2}-2\right)\right)\right]\right\}\\ & -H_{p_{2}}\left(p_{2}-2;\Delta_{d_{2}}\right)]. \end{array}$$

□

**Proof of Theorem 4.** We provide the proof of the ADR expressions of the proposed estimators. It is clear that

$$\lim_{n\to \infty }E\left[{\left\{n^{1/2}s_{1n}^{-1}d_{1n}^{T}\left({\hat{\beta }}_{1n}^{WR}-\beta_{1}\right)\right\}}^{2}\right]=E\left[\lim_{n\to \infty }{\left\{n^{1/2}s_{1n}^{-1}d_{1n}^{T}\left({\hat{\beta }}_{1n}^{WR}-\beta_{1}\right)\right\}}^{2}\right]=E\left[Z^{2}\right]=1,$$

where Z∼N(0,1). Then,

$$\begin{array}{cc} ADR(d_{1n}^{T}{\hat{\beta }}_{1n}^{RE}) & =\lim_{n\to \infty }E\left[{\left\{n^{1/2}s_{1n}^{-1}d_{1n}^{T}\left({\hat{\beta }}_{1n}^{RE}-\beta_{1}\right)\right\}}^{2}\right]\\ \; & =\lim_{n\to \infty }s_{1n}^{-2}E\left[{\left\{n^{1/2}d_{1n}^{T}\left[\left({\hat{\beta }}_{1n}^{WR}-\beta_{1}\right)-\left({\hat{\beta }}_{1n}^{WR}-{\hat{\beta }}_{1n}^{RE}\right)\right]\right\}}^{2}\right]\\ & =\lim_{n\to \infty }E\left[{\left\{n^{1/2}s_{1n}^{-1}d_{1n}^{T}\left({\hat{\beta }}_{1n}^{WR}-\beta_{1}\right)\right\}}^{2}\right]+\lim_{n\to \infty }E\left[{\left\{n^{1/2}s_{1n}^{-1}d_{1n}^{T}\left({\hat{\beta }}_{1n}^{WR}-{\hat{\beta }}_{1n}^{RE}\right)\right\}}^{2}\right]\\ \; & -2\lim_{n\to \infty }E\left[ns_{1n}^{-2}d_{1n}^{T}{\left({\hat{\beta }}_{1n}^{WR}-{\hat{\beta }}_{1n}^{RE})({\hat{\beta }}_{1n}^{WR}-\beta_{1}\right)}^{T}d_{1n}\right]\\ \; & =I_{1}+I_{2}+I_{3}. \end{array}$$

From (23), we have $I_{1}=\lim_{n\to \infty }E\left[{\left\{n^{1/2}s_{1n}^{-1}d_{1n}^{T}\left({\hat{\beta }}_{1n}^{WR}-\beta_{1}\right)\right\}}^{2}\right]=1$. Furthermore,

$$\begin{array}{cc} I_{2} & =\lim_{n\to \infty }s_{1n}^{-2}E{\left[n^{1/2}d_{1n}^{T}\left({\hat{\beta }}_{1n}^{WR}-{\hat{\beta }}_{1n}^{RE}\right)\right]}^{2}\\ \; & =\lim_{n\to \infty }\left(s_{2n}^{2}/s_{1n}^{2}\right)E{\left[n^{1/2}s_{2n}^{-1}d_{2n}^{T}{\hat{\beta }}_{2n}^{WR}\right]}^{2}. \end{array}$$

Since $s_{2n}^{2}/s_{1n}^{2}\to 1-c$, then,

$$I_{2}=\left(1-c\right)\lim_{n\to \infty }E\left[\chi_{1}^{2}\left(\Delta_{d_{2n}}\right)\right]=\left(1-c\right)\left(1+2\Delta_{d_{2}}\right).$$

Furthermore,

$$\begin{array}{cc} I_{3} & =-2\lim_{n\to \infty }E\left[ns_{1n}^{-2}d_{1n}^{T}\left({\hat{\beta }}_{1n}^{WR}-{\hat{\beta }}_{1n}^{RE}\right){\left({\hat{\beta }}_{1n}^{WR}-\beta_{1}\right)}^{T}d_{1n}\right]\\ \; & =2\lim_{n\to \infty }\left(s_{2n}/s_{1n}\right)E\left[n^{1/2}s_{2n}^{-1}d_{2n}^{T}{\hat{\beta }}_{2n}^{WR}n^{1/2}s_{1n}^{-1}{\left({\hat{\beta }}_{1n}^{WR}-\beta_{1}\right)}^{T}d_{1n}\right]\\ & =2{\left(1-c\right)}^{1/2}. \end{array}$$

Now, we investigate (25). By using Equation (17), we have

$$\begin{array}{cc} ADR(d_{1n}^{T}{\hat{\beta }}_{1n}^{SE}) & =\lim_{n\to \infty }E[\left\{n^{1/2}s_{1n}^{-1}d_{1n}^{T}{\left({\hat{\beta }}_{1n}^{SE}-\beta_{1})\right\}}^{2}\right]\\ \; & =\lim_{n\to \infty }E{\left[n^{1/2}s_{1n}^{-1}d_{1n}^{T}\left\{\left({\hat{\beta }}_{1n}^{WR}-\beta_{1}\right)-\left[\left(p_{2n}-2\right)/{\hat{T}}_{n}\right]\left({\hat{\beta }}_{1n}^{WR}-{\hat{\beta }}_{1n}^{RE}\right)\right\}\right]}^{2}\\ & =\lim_{n\to \infty }E{\left[n^{1/2}s_{1n}^{-1}d_{1n}^{T}\left({\hat{\beta }}_{1n}^{WR}-\beta_{1}\right)\right]}^{2}\\ & +\lim_{n\to \infty }E{\left[n^{1/2}s_{1n}^{-1}\left[\left(p_{2n}-2\right)T_{n}^{-1}\right]d_{1n}^{T}\left({\hat{\beta }}_{1n}^{WR}-{\hat{\beta }}_{1n}^{RE}\right)\right]}^{2}\\ \; & -2\lim_{n\to \infty }E\left[ns_{1n}^{-2}\left[\left(p_{2n}-2\right){\hat{T}}_{n}^{-1}\right]d_{1n}^{T}\left({\hat{\beta }}_{1n}^{WR}-{\hat{\beta }}_{1n}^{RE}\right){\left({\hat{\beta }}_{1n}^{WR}-\beta_{1}\right)}^{T}d_{1n}\right]\\ \; & =J_{1}+J_{2}+J_{3}. \end{array}$$

Again, $J_{1}=\lim_{n\to \infty }E\left[{\left\{n^{1/2}s_{1n}^{-1}d_{1n}^{T}\left({\hat{\beta }}_{1n}^{WR}-\beta_{1}\right)\right\}}^{2}\right]=1$. Then, we have

$$\begin{array}{cc} J_{2} & =\lim_{n\to \infty }E{\left[n^{1/2}s_{1n}^{-1}\left[\left(p_{2n}-2\right){\hat{T}}_{n}^{-1}\right]d_{1n}^{T}\left({\hat{\beta }}_{1n}^{WR}-{\hat{\beta }}_{1n}^{RE}\right)\right]}^{2}\\ & =\lim_{n\to \infty }{\left(p_{2n}-1\right)}^{2}E{\left[n^{1/2}s_{1n}^{-1}d_{2n}^{T}{\hat{\beta }}_{2n}^{WR}T_{n}^{-1}\right]}^{2}\\ & =\left(s_{2}^{2}/s_{1}^{2}\right){\left(p_{2}-1\right)}^{2}E{\left[\lim_{n\to \infty }\left\{n^{1/2}s_{2n}^{-1}d_{2n}^{T}{\hat{\beta }}_{2n}^{WR}{\hat{T}}_{n}^{-1}\right\}\right]}^{2}\\ & =\left(s_{2}^{2}/s_{1}^{2}\right){\left(p_{2}-1\right)}^{2}\left\{E\left[Z^{2}\chi_{p_{2}}^{-4}\left(\Delta_{d_{2}}\right)\right]+{\left(s_{2}^{-1}d_{2}^{T}\beta_{2}\right)}^{2}E\left[\chi_{p_{2}}^{-4}\left(\Delta_{d_{2}}\right)\right]\right\}\\ & =\left(1-c\right){\left(p_{2}-2\right)}^{2}\left\{E\left[\chi_{p_{2}+2}^{-4}\left(\Delta_{d_{2}}\right)\right]+{\left(s_{2}^{-1}d_{2}^{T}\beta_{2}\right)}^{2}E\left[\chi_{p_{2}}^{-4}\left(\Delta_{d_{2}}\right)\right]\right\}, \end{array}$$

and

$$\begin{array}{cc} J_{3} & =-2\lim_{n\to \infty }E\left[ns_{1n}^{-2}\left[\left(p_{2n}-2\right){\hat{T}}_{n}^{-1}\right]d_{1n}^{T}\left({\hat{\beta }}_{1n}^{WR}-{\hat{\beta }}_{1n}^{RE}\right){\left({\hat{\beta }}_{1n}^{WR}-\beta_{1}\right)}^{T}d_{1n}\right]\\ & =2\lim_{n\to \infty }\left(s_{2n}/s_{1n}\right)\left(p_{2n}-2\right)E\left[n^{1/2}s_{2n}^{-1}d_{2n}^{T}{\hat{\beta }}_{2n}^{WR}s_{1n}^{-1}{\left({\hat{\beta }}_{1n}^{WR}-\beta_{1}\right)}^{T}{\hat{T}}_{n}^{-1}\right]\\ & =2{\left(1-c\right)}^{1/2}\left(p_{2}-2\right)\left\{E\left[Z^{2}\chi_{p_{2}}^{-2}\left(\Delta_{d_{2}}\right)\right]+s_{2}^{-1}d_{2}^{T}\beta_{2}E\left[Z\chi_{p_{2}}^{-2}\left(\Delta_{d_{2}}\right)\right]\right\}\\ \; & =2{\left(1-c\right)}^{1/2}\left(p_{2}-2\right)E\left[\chi_{p_{2}+2}^{-2}\left(\Delta_{d_{2}}\right)\right]. \end{array}$$

Finally, we compute the ADR of ${\hat{\beta }}_{1n}^{PSE}$ as follows:

$$\begin{array}{cc} ADR(d_{1n}^{T}{\hat{\beta }}_{1n}^{PSE}) & =\lim_{n\to \infty }E\left[{\left\{n^{1/2}s_{1n}^{-1}d_{1n}^{T}\left({\hat{\beta }}_{1n}^{PSE}-\beta_{1}\right)\right\}}^{2}\right]\\ \; & =\lim_{n\to \infty }E[\{n^{1/2}s_{1n}^{-1}d_{1n}^{T}[\left({\hat{\beta }}_{1n}^{SE}-\beta_{1}\right)\\ & \;\;\;\;+\left[1-\left(p_{2n}-2\right){\hat{T}}_{n}^{-1}\right]\left({\hat{\beta }}_{1n}^{WR}-{\hat{\beta }}_{1n}^{RE}\right)I\left({\hat{T}}_{n}<\left(p_{2n}-2\right)\right)]{\}}^{2}]\\ & =\lim_{n\to \infty }E\left[{\left\{n^{1/2}s_{1n}^{-1}d_{1n}^{T}\left({\hat{\beta }}_{1n}^{SE}-\beta_{1}\right)\right\}}^{2}\right]\\ & +\lim_{n\to \infty }E\left[{\left\{n^{1/2}s_{1n}^{-1}d_{1n}^{T}\left[1-\left(p_{2n}-2\right){\hat{T}}_{n}^{-1}\right]\left({\hat{\beta }}_{1n}^{WR}-{\hat{\beta }}_{1n}^{RE}\right)I\left({\hat{T}}_{n}<\left(p_{2n}-2\right)\right)\right\}}^{2}\right]\\ \; & +2\lim_{n\to \infty }E[n^{1/2}s_{1n}^{-2}d_{1n}^{T}\left[1-\left(p_{2n}-2\right){\hat{T}}_{n}^{-1}\right]\left({\hat{\beta }}_{1n}^{WR}-{\hat{\beta }}_{1n}^{RE}\right){\left({\hat{\beta }}_{1n}^{SE}-\beta_{1}\right)}^{T}\\ & \;\;\;\times I\left({\hat{T}}_{n}<\left(p_{2n}-2\right)\right)d_{1n}]\\ & =ADR\left(d_{1n}^{T}{\hat{\beta }}_{1n}^{SE}\right)+\lim_{n\to \infty }E\left[{\left\{n^{1/2}s_{1n}^{-1}d_{1n}^{T}\left({\hat{\beta }}_{1n}^{WR}-{\hat{\beta }}_{1n}^{RE}\right)I\left({\hat{T}}_{n}<\left(p_{2n}-2\right)\right)\right\}}^{2}\right]\\ & +\lim_{n\to \infty }{\left(p_{2n}-2\right)}^{2}E[\left\{n^{1/2}s_{1n}^{-1}d_{1n}^{T}{\left({\hat{\beta }}_{1n}^{WR}-{\hat{\beta }}_{1n}^{RE}){\hat{T}}_{n}^{-1}I\left({\hat{T}}_{n}<\left(p_{2n}-2\right)\right)\right\}}^{2}\right]\\ & -2\lim_{n\to \infty }\left(p_{2n}-2\right)E\left[\left\{ns_{1n}^{-2}d_{1n}^{T}{\left({\hat{\beta }}_{1n}^{WR}-{\hat{\beta }}_{1n}^{RE}\right)}^{2}{\hat{T}}_{n}^{-1}I\left({\hat{T}}_{n}<\left(p_{2n}-2\right)\right)d_{1n}\right\}\right]\\ & +2\lim_{n\to \infty }E\left[\left\{ns_{1n}^{-2}d_{1n}^{T}\left({\hat{\beta }}_{1n}^{WR}-{\hat{\beta }}_{1n}^{RE}\right){\left({\hat{\beta }}_{1n}^{SE}-\beta_{1}\right)}^{T}I\left({\hat{T}}_{n}<\left(p_{2n}-2\right)\right)d_{1n}\right\}\right]\\ & -2\lim_{n\to \infty }\left(p_{2n}-2\right)E[\{ns_{1n}^{-2}d_{1n}^{T}\left({\hat{\beta }}_{1n}^{WR}-{\hat{\beta }}_{1n}^{RE}\right){\left({\hat{\beta }}_{1n}^{SE}-\beta_{1}\right)}^{T}\\ & \;\;\;\;\;\;\times {\hat{T}}_{n}^{-1}I\left({\hat{T}}_{n}<\left(p_{2n}-2\right)\right)d_{1n}\}]\\ & =ADR\left(d_{1n}^{T}{\hat{\beta }}_{1n}^{SE}\right)+K_{1}+K_{2}+K_{3}+K_{4}+K_{5}, \end{array}$$

where

$$\begin{array}{cc} K_{1} & =\lim_{n\to \infty }E[\left\{n^{1/2}s_{1n}^{-1}d_{1n}^{T}{\left({\hat{\beta }}_{1n}^{WR}-{\hat{\beta }}_{1n}^{RE})I\left({\hat{T}}_{n}<\left(p_{2n}-2\right)\right)\right\}}^{2}\right]\\ & =\lim_{n\to \infty }E\left[{\left\{n^{1/2}s_{2n}^{-1}d_{2n}^{T}{\hat{\beta }}_{2n}^{WR}I\left({\hat{T}}_{n}<\left(p_{2n}-2\right)\right)\right\}}^{2}\right]\\ & =\lim_{n\to \infty }{\left(s_{2n}/s_{1n}\right)}^{2}E\left[{\left\{n^{1/2}s_{2n}^{-1}d_{2n}^{T}{\hat{\beta }}_{2n}^{WR}I\left({\hat{T}}_{n}<\left(p_{2n}-2\right)\right)\right\}}^{2}\right]\\ & ={\left(s_{2}/s_{1}\right)}^{2}\left\{E\left[Z^{2}I\left(\chi_{p_{2}}^{2}\left(\Delta_{d_{2}}\right)<\left(p_{2}-2\right)\right)\right]+{\left(s_{2}^{-1}d_{2}^{T}\beta_{2}\right)}^{2}H_{p_{2}}\left(p_{2}-2;\Delta_{d_{2}}\right)\right\}\\ & =\left(1-c\right)\left\{E\left[\chi_{p_{2}+2}^{2}\left(\Delta_{d_{2}}\right)\right]+{\left(s_{2}^{-1}d_{2}^{T}\beta_{2}\right)}^{2}H_{p_{2}}\left(p_{2}-2;\Delta_{d_{2}}\right)\right\}, \end{array}$$

$$\begin{array}{cc} K_{2} & =\lim_{n\to \infty }E[\left\{n^{1/2}s_{1n}^{-1}d_{1n}^{T}{\left({\hat{\beta }}_{1n}^{WR}-{\hat{\beta }}_{1n}^{RE})\left(p_{2n}-2\right){\hat{T}}_{n}^{-1}I\left({\hat{T}}_{n}<\left(p_{2n}-2\right)\right)\right\}}^{2}\right]\\ & =\lim_{n\to \infty }{\left(p_{2n}-2\right)}^{2}{\left(s_{1n}/s_{2n}\right)}^{2}E\left[{\left\{n^{1/2}s_{2n}^{-1}d_{2n}^{T}{\hat{\beta }}_{2n}^{WR}{\hat{T}}_{n}^{-1}I\left({\hat{T}}_{n}<\left(p_{2n}-2\right)\right)\right\}}^{2}\right]\\ & ={\left(p_{2}-2\right)}^{2}{\left(s_{2}/s_{1}\right)}^{2}E\left[\lim_{n\to \infty }{\left\{n^{1/2}s_{2n}^{-1}d_{2n}^{T}{\hat{\beta }}_{2n}^{WR}{\hat{T}}_{n}^{-1}I\left({\hat{T}}_{n}<\left(p_{2n}-2\right)\right)\right\}}^{2}\right]\\ & ={\left(p_{2}-2\right)}^{2}\left(1-c\right)E\left[Z^{2}\chi_{p_{2}}^{-4}\left(\Delta_{d_{2}}\right)I\left(\chi_{p_{2}}^{2}\left(\Delta_{d_{2}}\right)<\left(p_{2}-2\right)\right)\right]\\ & ={\left(p_{2}-2\right)}^{2}\left(1-c\right)E\left[\chi_{p_{2}+2}^{-4}\left(\Delta_{d_{2}}\right)I\left(\chi_{p_{2}+2}^{2}\left(\Delta_{d_{2}}\right)<\left(p_{2}-2\right)\right)\right], \end{array}$$

$$\begin{array}{cc} K_{3} & =-2\lim_{n\to \infty }\left(p_{2n}-2\right)E[\left\{ns_{1n}^{-2}d_{1n}^{T}\left({\hat{\beta }}_{1n}^{WR}-{\hat{\beta }}_{1n}^{RE}{)}^{2}{\hat{T}}_{n}^{-1}I\left({\hat{T}}_{n}<\left(p_{2n}-2\right)\right)d_{1n}\right\}\right]\\ \; & =-2\left(p_{2}-2\right){\left(s_{2}/s_{1}\right)}^{2}E[\lim_{n\to \infty }{\left\{n^{1/2}s_{2n}^{-1}d_{1n}^{T}{\hat{\beta }}_{2n}^{WR}\right\}}^{2}{\hat{T}}_{n}^{-1}I\left({\hat{T}}_{n}<\left(p_{2n}-2\right)\right)]\\ & =-2\left(p_{2}-2\right)\left(1-c\right)\{E\left[Z^{2}\chi_{p_{2}}^{-2}\left(\Delta_{d_{2}}\right)I\left(\chi_{p_{2}}^{2}\left(\Delta_{d_{2}}\right)<\left(p_{2}-2\right)\right)\right]\\ & +{\left(s_{2}^{-1}d_{2}^{T}\beta_{2}\right)}^{2}E\left[\chi_{p_{2}}^{-2}\left(\Delta_{d_{2}}\right)I\left(\chi_{p_{2}}^{2}\left(\Delta_{d_{2}}\right)<\left(p_{2}-2\right)\right)\right]\}\\ & =-2\left(p_{2}-2\right)\left(1-c\right)\{E\left[\chi_{p_{2}+2}^{-2}\left(\Delta_{d_{2}}\right)I\left(\chi_{p_{2}+2}^{2}\left(\Delta_{d_{2}}\right)<\left(p_{2}-2\right)\right)\right]\\ & +{\left(s_{2}^{-1}d_{2}^{T}\beta_{2}\right)}^{2}E\left[\chi_{p_{2}}^{-2}\left(\Delta_{d_{2}}\right)I\left(\chi_{p_{2}}^{2}\left(\Delta_{d_{2}}\right)<\left(p_{2}-2\right)\right)\right]\}, \end{array}$$

$$\begin{array}{cc} K_{4} & =2\lim_{n\to \infty }E\left[\left\{ns_{1n}^{-2}d_{1n}^{T}\left({\hat{\beta }}_{1n}^{WR}-{\hat{\beta }}_{1n}^{RE}\right){\left({\hat{\beta }}_{1n}^{SE}-\beta_{1}\right)}^{T}I\left({\hat{T}}_{n}<\left(p_{2n}-2\right)\right)d_{1n}\right\}\right]\\ & =2\lim_{n\to \infty }\left(s_{2n}/s_{1n}\right)E\left[\left\{n^{1/2}s_{2n}^{-1}d_{2n}^{T}{\hat{\beta }}_{2n}^{WR}\right\}{\left\{n^{1/2}s_{1n}^{-1}d_{1n}^{T}\left({\hat{\beta }}_{1n}^{SE}-\beta_{1}\right)\right\}}^{T}I\left({\hat{T}}_{n}<\left(p_{2n}-2\right)\right)\right]\\ & =2\left(s_{2}/s_{1}\right)E[\left\{n^{1/2}s_{2n}^{-1}d_{2n}^{T}{\hat{\beta }}_{2n}^{WR}\right\}\{n^{1/2}s_{1n}^{-1}d_{1n}^{T}\left({\hat{\beta }}_{1n}^{WR}-\beta_{1}\right)\\ & -\left(p_{2n}-2\right){\hat{T}}_{n}^{-1}I{\left({\hat{T}}_{n}<\left(p_{2n}-2\right))\right\}}^{T}I\left({\hat{T}}_{n}<\left(p_{2n}-2\right)\right)]\\ & =2{\left(1-c\right)}^{1/2}\{E\left[\lim_{n\to \infty }\left\{n^{1/2}s_{2n}^{-1}d_{2n}^{T}{\hat{\beta }}_{2n}^{WR}\right\}{\left\{n^{1/2}s_{1n}^{-1}d_{1n}^{T}\left({\hat{\beta }}_{1n}^{WR}-\beta_{1}\right)I\left({\hat{T}}_{n}<\left(p_{2n}-2\right)\right)\right\}}^{T}\right]\\ & -\left(p_{2}-2\right)E\left[\left\{n^{1/2}s_{2n}^{-1}d_{2n}^{T}{\hat{\beta }}_{2n}^{WR}\right\}{\left\{n^{1/2}s_{1n}^{-1}d_{1n}^{T}\left({\hat{\beta }}_{1n}^{WR}-\beta_{1}\right){\hat{T}}_{n}^{-1}I\left({\hat{T}}_{n}<\left(p_{2n}-2\right)\right)\right\}}^{T}\right]\}\\ & =2{\left(1-c\right)}^{1/2}\left\{E\left[Z^{2}I\left(\chi_{p_{2}}^{2}\left(\Delta_{d_{2}}\right)<\left(p_{2}-2\right)\right)\right]-\left(p_{2}-2\right)E\left[Z^{2}\chi_{p_{2}}^{-2}\left(\Delta_{d_{2}}\right)I\left(\chi_{p_{2}}^{2}\left(\Delta_{d_{2}}\right)<\left(p_{2}-2\right)\right)\right]\right\}\\ & =2{\left(1-c\right)}^{1/2}\left\{H_{p_{2}+2}\left(p_{2}-2;\Delta_{d_{2}}\right)-\left(p_{2}-2\right)E\left[\chi_{p_{2}+2}^{-2}\left(\Delta_{d_{2}}\right)I\left(\chi_{p_{2}+2}^{2}\left(\Delta_{d_{2}}\right)<\left(p_{2}-2\right)\right)\right]\right\} \end{array}$$

and

$$\begin{array}{cc} K_{5} & =-2\lim_{n\to \infty }\left(p_{2n}-2\right)E\left[\left\{ns_{1n}^{-2}d_{1n}^{T}\left({\hat{\beta }}_{1n}^{WR}-{\hat{\beta }}_{1n}^{RE}\right){\left({\hat{\beta }}_{1n}^{SE}-\beta_{1}\right)}^{T}{\hat{T}}_{n}^{-1}I\left({\hat{T}}_{n}<\left(p_{2n}-2\right)\right)d_{1n}\right\}\right]\\ & =2\left(p_{2}-2\right)\left(s_{2}/s_{1}\right)E[\lim_{n\to \infty }\left\{n^{1/2}s_{2n}^{-1}d_{2n}^{T}{\hat{\beta }}_{2n}^{WR}{\hat{T}}_{n}^{-1}I\left({\hat{T}}_{n}<\left(p_{2n}-2\right)\right)\right\}\\ & \times {\left\{n^{1/2}s_{1n}^{-1}d_{1n}^{T}\left({\hat{\beta }}_{1n}^{WR}-\beta_{1}\right)-\left(p_{2n}-2\right)\left({\hat{\beta }}_{1n}^{WR}-{\hat{\beta }}_{1n}^{RE}\right){\hat{T}}_{n}^{-1}I\left({\hat{T}}_{n}<\left(p_{2n}-2\right)\right)\right\}}^{T}]\\ & =2\left(p_{2}-2\right)\left(s_{2}/s_{1}\right)\{E\left[Z^{2}\chi_{p_{2}}^{-2}\left(\Delta_{d_{2}}\right)I\left(\chi_{p_{2}}^{2}\left(\Delta_{d_{2}}\right)<\left(p_{2}-2\right)\right)\right]\\ & -\left(p_{2}-2\right)E\left[Z^{2}\chi_{p_{2}}^{-2}\left(\Delta_{d_{2}}\right)I\left(\chi_{p_{2}}^{2}\left(\Delta_{d_{2}}\right)<\left(p_{2}-2\right)\right)\right]\}\\ & =2\left(p_{2}-2\right){\left(1-c\right)}^{1/2}\{E\left[\chi_{p_{2}+2}^{-2}\left(\Delta_{d_{2}}\right)I\left(\chi_{p_{2}+2}^{2}\left(\Delta_{d_{2}}\right)<\left(p_{2}-2\right)\right)\right]\\ & -\left(p_{2}-2\right)E\left[\chi_{p_{2}+2}^{-4}\left(\Delta_{d_{2}}\right)I\left(\chi_{p_{2}+2}^{2}\left(\Delta_{d_{2}}\right)<\left(p_{2}-2\right)\right)\right]\}. \end{array}$$

□
